# Correction: Do women with a previous unintended birth subsequently experience missed opportunities for postpartum family planning counseling? A multilevel mixed effects analysis

**DOI:** 10.1371/journal.pgph.0004912

**Published:** 2025-07-09

**Authors:** Otobo I. Ujah, Jason L. Salemi, Rachel B. Rapkin, William M. Sappenfield, Ellen M. Daley, Russell S. Kirby

The fifth author’s name is spelled incorrectly. The correct name is: Ellen M. Daley. The correct citation is: Ujah OI, Salemi JL, Rapkin RB, Sappenfield WM, Daley EM, Kirby RS (2024) Do women with a previous unintended birth subsequently experience missed opportunities for postpartum family planning counseling? A multilevel mixed effects analysis. PLOS Glob Public Health 4(6): e0002570. https://doi.org/10.1371/journal.pgph.0002570
