# Supplementary material for: Do women with a previous unintended birth subsequently experience missed opportunities for postpartum family planning counseling? A multilevel mixed effects analysis
Source: PLOS Glob Public Health. 2024 Jun 5;4(6):e0002570. doi: 10.1371/journal.pgph.0002570 (PMC11152291; doi:10.1371/journal.pgph.0002570)
Supplement: Attachment — Submitted filename: PLOS GPH_Authors response.docx [file pgph.0002570.s002.docx]

**Author’s responses to reviewer’s comments**

Many thanks for kindly reviewing our manuscript. We have carefully considered all concerns raised and incorporated feedback, where possible. The quality of the revised manuscript has greatly improved as a result. Below we provide point-by-point responses to the observations raised.

This study undertakes a multilevel analysis of the association between the intendedness of a woman’s most recent pregnancy (in the past two years) and the likelihood of she experienced a missed opportunity for family planning (FP) counseling, using three cross-sectional rounds of PMA2020 survey data collected in Nigeria. The manuscript is well written, the methodology details are clear and properly interpreted. The research question warrants investigation. There are several measurement and confounding issues that need to be resolved.

1. **Missed opportunity for family planning counseling**

The outcome variable is similar to that studied by Thiongo et al. (2022, reference #24) but the Thiongo study does not test a relationship with pregnancy wantedness (the exposure variable). The characterization of a “missed opportunity” is the analyst’s judgment, not an experience directly reported by the female survey respondent. The outcome is based on the female respondent’s report of either 1) visiting a health facility within the past 12 months where the provider spoke to her about family planning or 2) being visited by a community health worker who spoke to her about FP. If neither happens, then the outcome is defined as a missed opportunity.

Is this definition really “missed”, however? Two issues here. First, in this study there is no examination of whether the woman has begun to use contraception postpartum, an outcome examined in the Thiongo paper. It showed for Kenya that 61% of their sample (also women who gave birth in the past 24 months) were using contraception at 6-11 months postpartum and 66% at 12-23 months. While contraceptive use is less prevalent in Nigeria than in Kenya, the inclusion of women who may be contracepting postpartum, nonetheless, is important to avoid misplaced inference. A woman could have adopted a method (such as a postpartum IUD) in the first month after birth, or she could have obtained a contraceptive injectable (or pill or condom) from a non-clinical provider (pharmacy) and not perceive or report this as a health facility visit.

Second, the “missed” opportunity experience does not address the strong likelihood of a family planning discussion during antenatal visits, which has high coverage in Nigeria. The intendedness of that pregnancy is the same one being linked to a postpartum missed opportunity.

In the present study, the authors should exclude postpartum women who adopted contraception after birth from the sample. Alternatively they could add contraceptive use at 24 months as a second outcome. If they pursue the latter suggestion, the analytic approach could be either current use status or time to adoption (requiring multivariable hazard regression modeling).

It is recommended that “Postpartum” be inserted before “Family Planning Counseling” in the title, since any health provider encounter that does not elicit a contraceptive counseling discussion for the pregnant woman is a missed opportunity.

Given that the outcome distribution is around 50%, is a logit function the most appropriate form to model? Would a probit regression be better? The results likely will not be very different but with the probit function, you could calculate average marginal effects and discuss the findings as predicted probabilities which are more easily understood than adjusted odds ratios.

**Author’s response**

1. Thank you for this important observation. Accordingly, we have excluded postpartum women who were using contraception after birth from the analytic sample to avoid misleading inferences based on the results of our study.
2. We have also included postpartum in the title of the paper so it no reads “Postpartum Family Planning”.
3. Regarding the choice of using the probit or logit link function, we acknowledge that probit regression is an alternative option. However, due to computational efficiency and widespread use in similar studies, we opted to use logit function. Additionally, exponentials of the logit regression coefficients are readily interpretable as odds ratios, facilitating a straightforward understanding of the impact of pregnancy intendedness and the other independent predictors on the odds of MOs by readers. Moreover, with the exclusion of the sample of women using a contraceptive method after birth, the outcome distribution is no longer around 50%.
2. **Pregnancy wantedness/intendedness**

The present study’s exposure variable is the intended status of the woman’s most recent pregnancy. The female respondent reports this intendedness at the time of the survey although the pregnancy could have occurred up to 33 months before the survey. This is because women giving birth (not conceiving) in the past 24 months are in the sample. This raises a temporal order issue with the missed opportunity outcome, since the woman can visit a health facility another reason (a sick child) that wouldn’t invoke a discussion of family planning need.

The inclusion of fertility preferences as a control variable, along with pregnancy intendedness as the exposure variable, also raises some concern. Fertility preferences refer to the future but will be strongly correlated with pregnancy intendedness. The authors should investigate the interactions of these two variables and assess whether the preferences variable warrants being retained.

**Author’s response**

We do acknowledge the temporal order issue raised concerning the exposure variable as a poses a considerable limitation to the findings of our study, especially with the cross-sectional design of our study. We have highlighted this in the limitation section of our study.

We also agree that fertility preferences will be strongly correlated with pregnancy intendedness and therefore have excluded this variable in the analysis for the revised manuscript.

3. **Cross-sectional versus longitudinal PMA survey data**

The cross-sectional nature of the PMA2020 survey data constrains the authors’ ability to test the main hypothesis. PMA survey data (pmadata.org) which succeeded PMA2020 are now longitudinal, i.e., panels of women are followed from one annual round to the next. Although the Nigeria data ae limited to two states (Lagos and Kano), there ae other national surveys with up to three panel rounds (i.e., Burkina Faso, Cote d’Ivoire, Kenya, Uganda) whose data are publicly available. The advantage of using panel data obviously is that the reported intendedness of the most recent birth within 12 months of the baseline round can be associated with the same woman’s report of any health visit with or without family planning counseling in the second round and third round. Adoption of contraception in these rounds can be treated as a competing or secondary outcome. Using longitudinal data will strengthen causal inference from findings about the relationships of interest without temporal confounding.

**Author’s response**

We do acknowledge that the use of cross-sectional data limits our ability to draw causal inferences. The primary goal of this study was to generate nationally representative estimates for Nigeria and not to establish causality. Therefore, at the time of our analyses, the PMA cross-sectional survey data were the only available data that could enable us to generate nationally representative estimates, as opposed to using longitudinal data from specific regions like Lagos and Kano, which would have offered only subnational estimates. Also, the scope of our research centers on Nigeria and nationally representative samples, precluding the inclusion of data from other countries. Despite this limitation, our findings can provide valuable insights for guiding future studies that may be willing to leverage existing longitudinal PMA survey data for more robust analyses.

4. **Theoretical framework**

The Traits-Desire-Intention-Behavior framework informs this analysis. The Discussion should return to considering how the findings enlighten the applicability of the TDIB framework. The outcome behavior, non-receipt of contraceptive counseling, in this case, is not one chosen by the individual woman but an experience inferred for her. Contraceptive utilization as an outcome behavior, on the other hand, would be. What do the findings regarding contextual effects say about the framework?

**Author’s response**

We have revised the manuscript to highlight the applicability of the TDIB framework in explaining MOs for postpartum FP counseling in the discussion section.

5. **Tertile gradients for community-level variables**

The community-level variables are categorized into tertiles. What is the justification for this? Using artificial cut-points like this can mitigate the variation with the other covariates and outcome variable. Did the author(s) examine the distributions for empirical cut-off points or try quintiles?

Author’s response

Our initial approach to using tertiles was based on a previous study by OlaOlorun et al. (2020). However, with the concern raised regarding mitigation of the variation, we have operationalized these community-level variables as continuous variables which were grand-mean centered for the multilevel analysis.

**Reference**

OlaOlorun, F. M., Anglewicz, P., & Moreau, C. (2020). From non-use to covert and overt use of contraception: Identifying community and individual factors informing Nigerian women’s degree of contraceptive empowerment. *PLOS ONE*, *15*(11), e0242345. <https://doi.org/10.1371/journal.pone.0242345>

Minor comments

1. Not a concern but a compliment on building a hierarchical model with cluster aggregated measures. The diagnostic results clearly reveal the inter-cluster variability in Nigeria and its contribution to explaining variation in the outcome.

**Author’s response**

Thank you.

2. These 2016-2018 surveys originated under the PMA2020 program, whereas the present program is called just “PMA”. Would suggest relabeling them in this study.

**Author’s response**

We have relabeled the surveys to now read PMA2020 in the revised manuscript.

3. P 9, second line from bottom – why is this a “subsample” and not the “total sample”?

**Author’s response**

We have clarified this in the revised manuscript and now reads “total sample” (pg 10).

4. What is the justification for pooling 3 rounds – sample power or to isolate secular change by survey year? Should explain.

**Author response**

We have provided an explanation of the rationale for pooling data from 3 rounds of the PMA2020 survey. In the revised manuscript, we state that “**By p**ooling data from multiple survey rounds, our goal was to ensure a larger sample size and ultimately, increase the statistical power of the current study.” (pg 10).

5. P. 13, line 4 – the text is not clear.

**Author response**

We clarified this in the revised manuscript to avoid ambiguity.

6. P. 14, p_ij_ needs to be defined, as π*ij /*1-π*ij*.

**Author response**

We defined this in the revised manuscript (pg 14).

Logit(*p*_ij_) = log[*π_ij_/*1-*π_ij_*] = *β*_0_ + *β*_1_X_1_*_ij_* + … + *β_n_*X*_nij_* + *ʋ*_0_*_j_*  + *ɛ_ij,_*

Where: The logit of *p_ij_* is denoted as a sum of linear function of the independent variables.

7. Throughout the manuscript and in the tables, be consistent in the use of decimal places, either 1 or 2.

**Author response**

In the revised manuscript and in the tables, we ensured that estimates are now consistently rounded to 1 decimal place.

8. P. 37, para 1, line 7-8, sentence not clear.

**Author response**

We clarified this in the revised manuscript to avoid ambiguity.
