# Supplementary material for: Do women with a previous unintended birth subsequently experience missed opportunities for postpartum family planning counseling? A multilevel mixed effects analysis
Source: PLOS Glob Public Health. 2024 Jun 5;4(6):e0002570. doi: 10.1371/journal.pgph.0002570 (PMC11152291; doi:10.1371/journal.pgph.0002570)
Supplement: Attachment — Submitted filename: Comments on revised manuscript.docx [file pgph.0002570.s003.docx]

Thank you for the changes and improved manuscript. There are still some small concerns that need to be addressed but overall the analysis is on sounder ground.

1. Table 1 – I believe footnotes a and b need to be reversed.
2. Please clarify – on p. 18 the text refers to a sample size of 6,479 women and 822 clusters, while on p. 16 the number of clusters is 420. Tables 2 and 3 cite 822 clusters. Is 420 a typo? PMA2020’s sample design involved repeated use of clusters in the first 4 survey rounds (which were about 6 months apart), followed by a new selection of sample clusters for rounds 5 and 6 but that number would have been the same as those in rounds 1-4.
3. Please do a quick search for the use of “determinants” and replace with “factors associated with”. “Determinants” should be reserved for use with causal analyses.
4. On p. 17, the text (3 lines from bottom) says “nearly two thirds (58.5%)”; this should be “nearly three-fifths”, since 58.5% is closer to 60% than to 67%.
5. On p. 34, the narrative on limitations states, “Second, the ascertainment of MOs was based on women’s self-report.” This is not the case and was the point of my first comment, i.e., that a missed opportunity is operationally defined by the study researchers but is not self-reported by the female respondent. The woman was not directly asked if she experienced a missed opportunity for FP counseling. For example, she could have been asked if she was interested in receiving FP information after her delivery and, if so, where/when would have been the most appropriate time/place. From a health policy and service coverage perspective, all postpartum clients assessed by providers to have a contraceptive need should be offered services, and this is what is driving the researchers’ definition and study interests. Please adjust the wording here.
6. Last, just to underscore the importance of the cluster-level findings, I think these reflect a normative climate In Nigeria that does not support FP usage in general. Thus providers will not think to mention FP to PP clients or may not diligently follow MCH protocol to screen for past unplanned pregnancies. Mothers may be hesitant to ask about pregnancy prevention means in a public facility setting. I recommend the Discussion emphasize the need for further study of community attitudes and norms about FP that influence providers’ and mothers’ attitudes and comfort to receipt of such information after delivery.
