# Supplementary material for: Do women with a previous unintended birth subsequently experience missed opportunities for postpartum family planning counseling? A multilevel mixed effects analysis
Source: PLOS Glob Public Health. 2024 Jun 5;4(6):e0002570. doi: 10.1371/journal.pgph.0002570 (PMC11152291; doi:10.1371/journal.pgph.0002570)
Supplement: Attachment — Submitted filename: Authors response v2.docx [file pgph.0002570.s004.docx]

**Rebuttal Letter**

**Original Manuscript ID:** PGPH-D-23-01986

**Article Title: “**Do Women with a Previous Unintended Birth Subsequently Experience Missed Opportunities for Postpartum Family Planning Counseling? A Multilevel Mixed Effects Analysis”

**To:** PLOS Global Public Health

**Re:** Response to reviewers

Dear Editor,

Thank you for allowing a resubmission of our revised manuscript, with an opportunity to address the reviewers’ comments.

Herein, we upload

(1) Our point-to-point response to the comments below (response to reviewer(s) and academic editor, respectively)

(2) A marked-up coy with track changes (Revised Manuscript with Track Changes)

(3) An unmarked version without track changes (Manuscript)

Journal Requirements:
Please review your reference list to ensure that it is complete and correct. If you have cited papers that have been retracted, please include the rationale for doing so in the manuscript text, or remove these references and replace them with relevant current references. Any changes to the reference list should be mentioned in the rebuttal letter that accompanies your revised manuscript. If you need to cite a retracted article, indicate the article’s retracted status in the References list and also include a citation and full reference for the retraction notice.

**Author’s response**: **Response: The reference list is complete and correct. No retracted article has been cited.**

**Reviewer #1**

Thank you for the changes and improved manuscript. There are still some small concerns that need to be addressed but overall the analysis is on sounder ground.

1. Table 1 – I believe footnotes a and b need to be reversed.

**Author’s response: Thank you for spotting this error. We have reversed this in the revised manuscript.**

2. Please clarify – on p. 18 the text refers to a sample size of 6,479 women and 822 clusters, while on p. 16 the number of clusters is 420. Tables 2 and 3 cite 822 clusters. Is 420 a typo? PMA2020’s sample design involved repeated use of clusters in the first 4 survey rounds (which were about 6 months apart), followed by a new selection of sample clusters for rounds 5 and 6 but that number would have been the same as those in rounds 1-4.

**Author’s response: Thank you for this important observation. Yes, 420 is a typo. For the overall sample, it should be 6,479 women nested in 877 clusters and**

**nested in 420 clusters. For the subsample of women who visited a health facility in the past 12 months, it should be 4,194 women nested in 822. We have corrected this in the revised manuscript.**

3. Please do a quick search for the use of “determinants” and replace with “factors associated with”. “Determinants” should be reserved for use with causal analyses.

**Author’s response: We have replaced “determinants with “factors associated with”**

4. On p. 17, the text (3 lines from bottom) says “nearly two thirds (58.5%)”; this should be “nearly three-fifths”, since 58.5% is closer to 60% than to 67%.

**Author’s response: We have changed this to “Nearly 60% experienced a MOs for postpartum FP counseling during health facility and/or CHW visits”.**

5. On p. 34, the narrative on limitations states, “Second, the ascertainment of MOs was based on women’s self-report.” This is not the case and was the point of my first comment, i.e., that a missed opportunity is operationally defined by the study researchers but is not self-reported by the female respondent. The woman was not directly asked if she experienced a missed opportunity for FP counseling. For example, she could have been asked if she was interested in receiving FP information after her delivery and, if so, where/when would have been the most appropriate time/place. From a health policy and service coverage perspective, all postpartum clients assessed by providers to have a contraceptive need should be offered services, and this is what is driving the researchers’ definition and study interests. Please adjust the wording here.

**Author’s response: We have adjusted the wording in the revised manuscript. This is contained in pages 33-34.**

6. Last, just to underscore the importance of the cluster-level findings, I think these reflect a normative climate In Nigeria that does not support FP usage in general. Thus providers will not think to mention FP to PP clients or may not diligently follow MCH protocol to screen for past unplanned pregnancies. Mothers may be hesitant to ask about pregnancy prevention means in a public facility setting. I recommend the Discussion emphasize the need for further study of community attitudes and norms about FP that influence providers’ and mothers’ attitudes and comfort to receipt of such information after delivery.

**Author’s response: Thank you for these helpful comments. We have included these in the revised manuscript as contained on page 35.**

**Reviewer #2**

Comment: This revision is well executed. The authors have handsomely addressed all the issues in the manuscript. However, there are little issues with the introduction. I think it will be more appropriate if the authors delete either the questions or the objectives. Both research questions or objectives indicate double standards in the manuscript. I suggest that the authors delete the research questions as the research objectives of the study are well-defined and written.

**Author’s response: Thank you for this suggestion. We have deleted the research questions, leaving only the research objectives in the revised manuscript.**
